# Self-Powered Room-Temperature Ethanol Sensor Based on Brush-Shaped Triboelectric Nanogenerator

**DOI:** 10.34133/2021/8564780

**Published:** 2021-03-01

**Authors:** Jingwen Tian, Fan Wang, Yafei Ding, Rui Lei, Yuxiang Shi, Xinglin Tao, Shuyao Li, Ya Yang, Xiangyu Chen

**Affiliations:** ^1^CAS Center for Excellence in Nanoscience, Beijing Key Laboratory of Micro-Nano Energy and Sensor, Beijing Institute of Nanoenergy and Nanosystems, Chinese Academy of Sciences, Beijing 100083, China; ^2^School of Nanoscience and Technology, University of Chinese Academy of Sciences, Beijing 100049, China

## Abstract

Highly sensitive ethanol sensors have been widely utilized in environmental protection, industrial monitoring, and drink-driving tests. In this work, a fully self-powered ethanol detector operating at room temperature has been developed based on a triboelectric nanogenerator (TENG). The gas-sensitive oxide semiconductor is selected as the sensory component for the ethanol detection, while the resistance change of the oxide semiconductor can well match the “linear” region of the load characteristic curve of TENG. Hence, the output signal of TENG can directly reveal the concentration change of ethanol gas. An accelerator gearbox is applied to support the operation of the TENG, and the concentration change of ethanol gas can be visualized on the Liquid Crystal Display. This fully self-powered ethanol detector has excellent durability, low fabrication cost, and high selectivity of 5 ppm. Therefore, the ethanol detector based on TENG not only provides a different approach for the gas detection but also further demonstrates the application potential of TENG for various sensory devices.

## 1. Introduction

As a colorless and transparent volatile chemical liquid commonly found in industrial products, the administering and monitoring of ethanol is crucial for industrial safety and drink-driving tests [1–3]. An effective and convenient ethanol detector can avoid potential economic loss and casualties caused by ethanol abuse. The conventional energy supply for a gas detector is mostly relying on lithium-ion batteries. Accordingly, the batteries should be replaced or charged frequently, and the exhausted batteries may also bring pollution risks to the environment and the human body [4, 5]. Moreover, commercial gas sensors are generally working at a high temperature, in order to maintain the high sensitivity, which may further increase the energy consumption. According to operating characteristics and sensitive mechanisms, gas sensors can be classified into different categories, including solid electrolyte [6], semiconductor, contact combustion [7], and electrochemical [8]. Among all these devices, semiconductor gas sensors have been widely focused on because of advantages of real-time detection, simple structure, high sensitivity, and all-solid state [9]. For instance, tungsten trioxide (WO_3_) is one resistive semiconductor material that has been widely used in gas sensors, humidity sensors, and other fields due to nontoxicity and stability [10].

On the other hand, TENG has been recognized as a safe, low-cost, and portable source of energy supply in recent years on account of contact electrification and electrostatic induction [11, 12]. Compared to commercial power sources, materials of TENG are widely available and inexpensive. Besides, the design of TENG can be adapted to different structural parameters to obtain the desired electrical properties [13–15]. The self-powered gas sensor is one of the promising application directions of TENG. Most of these sensors are relying on the interaction effect between the triboelectric interface and gas molecules, where the surface charge density of the TENG is influenced by the atmosphere of the medium. For instance, Cui et al. have proposed a self-powered ammonia nanosensor, and the polyaniline nanofibers with NH_3_ sensing property acts as the electrification layer of TENG [16]. These kinds of TENG-based sensors are generally less sensitive than those using resistive semiconductor materials, and the repeatability of the electrification interface for gas detection is also a challenge. Alternatively, using the energy from TENG to directly drive semiconductor sensors can be another approach for achieving high sensitivity. Wen et al. have studied this kind of combined gas sensor [17], where TENG serves as a pure power source. However, the devices cannot work at the room temperature, which means the heater may consume additional energy.

Here, a fully self-powered ethanol detector based on TENG and the WO_3_ gas sensor is proposed, which can visualize the concentration change of ethanol at room temperature. With the structural modification, the “linear” region of the voltage-load curve of TENG can well match with the resistance changing of the WO_3_ component. Accordingly, the concentration change of ethanol in a range of 5-100 parts per million (ppm) can be detected by the output signal of TENG. Two Liquid Crystal Displays (LCDs) with different driving voltages are integrated with TENG, where the output voltage of TENG can selectively control different LCDs and results in the visualization of the concentration change. This self-powered detector has good gas response after two weeks in the air environment, and it exhibits excellent selectivity for ethanol gas. In view of the distinctive working mechanism and novel structural design, this self-powered ethanol detector has a series of excellent properties such as lower detection limit, room-temperature working conditions, and a fully self-powered system. This work not only has certain reference significance in the field of gas sensors but also further expands the applicability of TENGs as a self-powered sensor.

## 2. Results and Discussion

**Figure 1:**
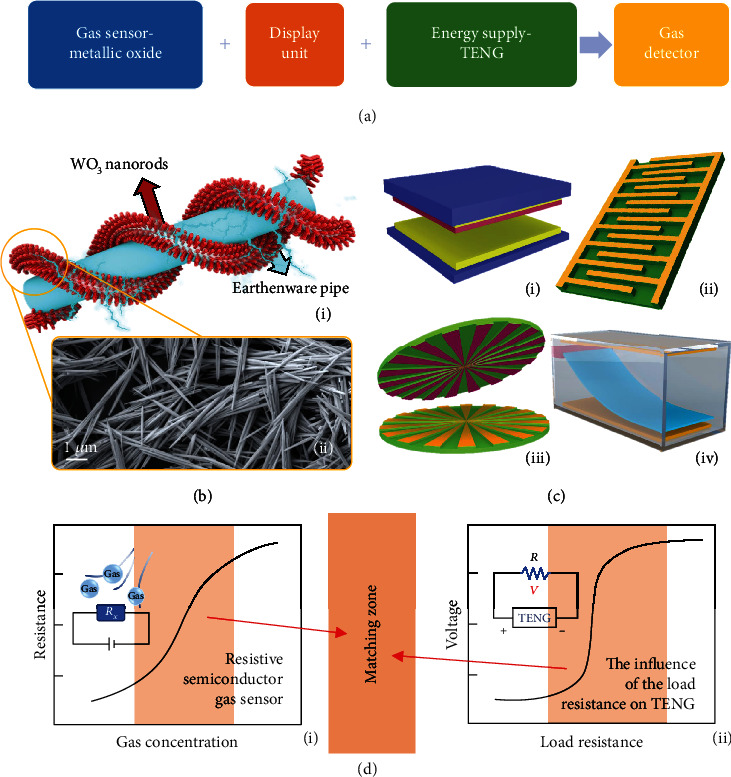
Schematic diagram of the self-powered room-temperature ethanol detection sensor. (a) The conception of the self-powered sensing system composed of 3 parts. (b) Schematic diagram of WO_3_ gas sensor (i) and the SEM image of the WO_3_ (ii). (c) Four representative TENG structures, vertical contact-separation mode (i), contact-sliding mode (ii), freestanding triboelectric-layer mode (iii), and single-electrode mode (iv). (d) The characteristic curve of a p-type semiconductor gas-sensitive material exposed to reducing gases and the measurement circuit diagram (i). A characteristic curve of voltage when various external resistances are connected to the TENG and the system circuit measurement (ii).

The design concept of the self-powered ethanol sensor mainly consists of three parts: gas sensor, display units, and the energy supply (see Figure 1(a)). The semiconductor metal oxide of WO_3_ is selected as the core element of the gas sensor, which depends on its advantages such as straightforward manufacture, convenient operation, low cost, and small volume. WO_3_ nanorods can be synthesized as a kind of p-type semiconductor under the reaction conditions shown in the experimental part. As shown in Figure 1(b), (i), WO_3_ nanorods are painted on the surface of the earthenware tube to enlarge the effective surface area for gas detection. Under the scanning electron microscope (SEM), the morphologic structure of WO_3_ nanorods can be seen in Figure 1(b), (ii). The synthesized WO_3_ nanorods have excellent dispersion and a large specific surface area, which is the basis for obtaining high gas sensitivity. In this work, TENG has been employed as the power source to drive the whole detector system. There are various TENG structures which can be selected for this purpose, and we summarize four kinds of typical TENGs, as can be seen in Figure 1(c), which are vertical contact-separation mode (Figure 1(c), (i)), contact-sliding mode (Figure 1(c), (ii)), freestanding triboelectric-layer mode (Figure 1(c), (iii)), and single-electrode mode (Figure 1(c), (iv)) [18–21]. Due to variable parameters, TENG is flexible to be designed with different electrical properties, which can make it suitable for a variety of circumstances [22–24]. Resistance matching between the sensory oxide semiconductor and TENG is very crucial for this system. In Figure 1(d), the characteristic curve of a p-type semiconductor (WO_3_) gas-sensitive material exposed to reducing gases has been shown. The resistance of the semiconductor material increases gradually with the increment of gas concentration, especially in the orange region (the measurement circuit diagram is shown in Figure 1(d)). Meanwhile, when TENG is externally coupled with a load resistance *R*, its output voltage is stable at first and then gradually increases to a certain value with the increase of *R* (the system circuit measurement is shown in Figure 1(d), (ii)), leading to a characteristic curve of voltage in Figure 1(d), (ii). The output voltage-load resistance graphs of almost all TENGs always show this kind of curve with three work areas. The first working area is related to small *R*, where the output voltage is almost unchanged compared with the short circuit. The second working area is the “linear” region that the output voltage increases rapidly with *R*. As *R* gradually increases to a certain value, the output voltage reaches the saturation. Therefore, the key point of this detector is to adjust the parameters of both TENG and gas sensor and to achieve the “matching zone” shown in Figure 1(d), which can associate the characteristic properties of TENG and gas-sensitive materials.

**Figure 2:**
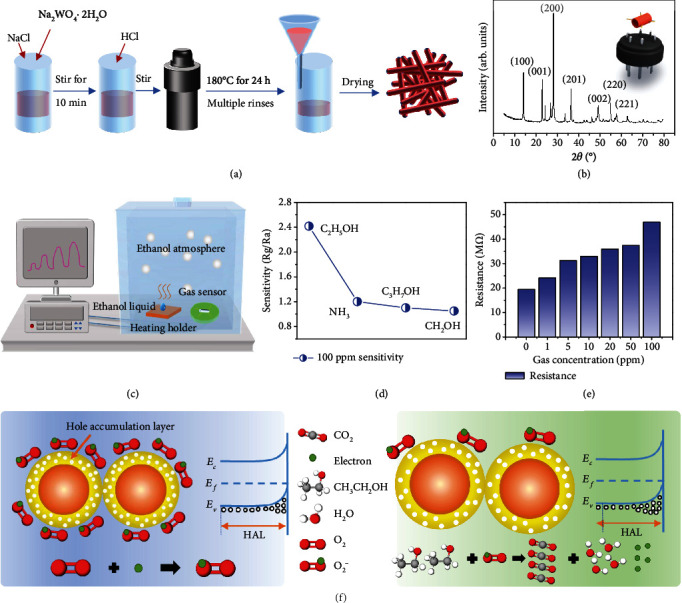
(a) A typical procedure of the hydrothermal technology to synthesize WO_3_ nanorods. (b) The XRD pattern of WO_3_ nanorods and the schematic diagram of the assembled gas sensor. (c) A schematic diagram of the measurement system. (d) The selectivity of the gas sensor for different types of gas detection. (e) The dynamic gas response of the WO_3_ gas sensor at different concentrations of 1 ppm to 100 ppm. (f) The schematic diagram of the WO_3_ gas-sensing mechanism and the schematic diagram of hole carrier conduction.

There are many common gas detection materials, such as MoO_3_ [25, 26], ZnO [27, 28], and TiO_2_ [29, 30]. However, p-type semiconductors for gas sensing should be given priority, because the external load resistance of TENG is positively correlated with its output voltage. At the same time, the range of resistance in TENG areas needs to be matched with resistance of gas-sensitive materials, which should be in the scale of M*Ω*. Besides, the working condition of room temperature also influences the working temperature of gas-sensitive materials. In conclusion, WO_3_ has been selected as the material for the gas sensor. The hydrothermal preparation process is applied to synthesized WO_3_ nanorods, a typical procedure of which is shown in Figure 2(a). Firstly, Na_2_WO_4_·2H_2_O and NaCl are placed into deionized (DI) water, and the mixture is stirred continuously for 10 minutes until it dissolves. Among them, Na_2_WO_4_·2H_2_O is prepared as a precursor and NaCl acts as a structure guiding agent. At the nanoscale, NaCl has been demonstrated to promote the growth of WO_3_. Subsequently, HCl is added gradually to adjust the pH of the mixture to acidic conditions. The pH can maintain the proton-deprotonation balance of hydroxylated groups on the surface of the growth unit. As shown in Figure S1, the hydrothermal reaction can obtain diverse WO_3_ with morphologies under different pH values. When the pH value is between 1.7 and 2.3, the agglomeration of hydrothermal products is serious. At a pH of 2.5, a large number of uniformly dispersed WO_3_ nanorods have been synthesized. The directions of these nanorods are random, indicating that there is no significant periodicity in the arrangement and that they are separated from each other with the length from 2 to 5 *μ*m. WO_3_ with good dispersion has a larger specific surface area, which means it can provide more active sites for gas molecules, which is conducive to the detection of target gas and improves the sensitivity of gas detection. At the same time, reducing the agglomeration of WO_3_ materials can improve the long-term stability of gas sensors and result in a gas sensor with uniform gas-sensitive properties. [31] However, no precipitate has been found in the sample prepared at a pH value of 3, which can be explained by the disappearance of H_2_WO_4_ according to the synthesis principle of WO_3_ nanorods. Hence, the pH value of this study is fixed at 2.5. Next, the mixture is transferred to a Teflon-lined stainless steel autoclave and kept at 180°C for 24 h. The mixture is then cooled at room temperature and washed alternately with DI water and ethanol. The filtered solid powder of WO_3_ is then dried in an oven. After being uniformly ground with DI, the slurry is coated on an earthenware tube. Finally, the earthenware tube is soldered to the base. The upper right corner of Figure 2(b) shows the diagram of the assembled gas sensor. At the same time, the EDS analysis of Figure S2 shows that the atomic ratio of O and W is close to 3 : 1. The presence of the C element can be considered as the presence of the conductive tapes. The main diffraction peaks of the obtained WO_3_ can be indicated as a hexagonal WO_3_ with lattice parameters.

Figure 2(c) shows the measurement system. The gas sensitivity test was performed at a room temperature of 20.5°C and humidity of 24%RH. The gas sensors are placed in an acrylic enclosed container at room temperature. The ethanol liquid is injected on a PTC thermostatic heater, which evaporated liquid into gas for measurement. Figure 2(d) shows the selectivity of the gas sensor for different gas detection. The different gas response of the WO_3_ gas sensor to 100 parts per million (ppm) of NH_3_ (1.20), C_3_H_7_OH (1.10), and CH_3_OH (1.05) exhibits that the sensor is selective for C_2_H_5_OH (2.41) at room temperature. In the case of ethanol, the dynamic gas response of the WO_3_ gas sensor at different concentrations of 1 to 100 ppm has been examined, as shown in Figure 2(e). The response resistance gradually increased from 19.4 M*Ω* to 47 M*Ω* when WO_3_ sensitive materials are exposed to ethanol, which is an obvious p-type semiconductor feature.

The schematic diagram of WO_3_ gas detection is explained in Figure 2(f). Generally, the conductivity of the p-type semiconductor is mainly affected by hole carrier concentration and mobility. When a gas-sensitive material is placed in the air, a certain amount of oxygen molecules is adsorbed on the surface. The surface adsorbed oxygen shows different forms (O^2−^, O^−^, and O_2_^−^) at different temperatures, and O_2_^−^ is mainly at room temperature [32, 33].

(1) $$\begin{array}{c} \left(1\right)\;{\text{O}}_{2}\;\left(\text{gas}\right)\Leftrightarrow {\text{O}}_{2}\;\left(\text{absorbed}\right)\\ \left(2\right)\;{\text{O}}_{2}\;\left(\text{absorbed}\right)+{\text{e}}^{-}\Leftrightarrow {{\text{O}}_{2}}^{-}\;\left(\text{below }100^{{}^{\circ}}\text{C}\right)\\ \left(3\right)\;{{\text{O}}_{2}}^{-}+{\text{e}}^{-}\Leftrightarrow 2{\text{O}}^{-}\;\left(100\text{ to }300^{{}^{\circ}}\text{C}\right)\\ \left(4\right)\;{\text{O}}^{-}+{\text{e}}^{-}\Leftrightarrow {\text{O}}^{2-}\;\left(\text{above }300^{{}^{\circ}}\text{C}\right) \end{array}$$

According to the electrostatic interaction between the opposite electrical charges, the adsorption of O_2_^−^ in p-type WO_3_ forms the hole-accumulation lay (HAL) on the near surface. The HAL leads to the increase of carrier concentration in the material and, thus, leads to the increase of material conductivity [8]. When a p-type sensitive material comes into contact with a reducing gas, the gas molecules react with the oxygen adsorbed on the surface of the material, causing the electrons trapped by the oxygen molecules to return to the conductive band of the sensitive material. In this process, carrier concentration decreases, and the width of the space depletion layer increases, leading to the decrease of material conductivity. In addition, the WO_3_ gas sensor has certain anti-interference and durability. After two weeks of natural storage at room temperature, the baseline resistance of the WO_3_ gas sensor rose slightly to 35 M*Ω* but still responded well to ethanol. As shown in Figure S3, 100 ppm ethanol can make the resistance value of the gas sensor change by about 20 M*Ω*.

**Figure 3:**
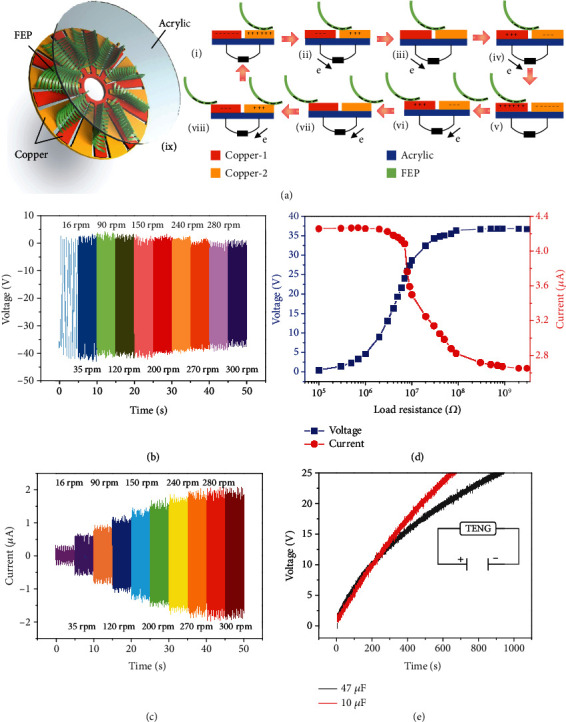
(a) The schematic diagram of the basic structure and the working principle of the BS-TENG. Electrical output characterizations of the BS-TENG. Dependence of the (b) open-circuit voltage and (c) short-circuit current on various rotating speeds from 16 to 300 rpm. (d) The variation of the output voltage and current with external load resistances under the rotating speed of 300 rpm. (e) A 10 *μ*F and a 47 *μ*F capacitor charged by BS-TENG under the rotating speed of 300 rpm. The 47 *μ*F capacitor can be charged to 25 V in 15 min from the initial state.

According to different application scenarios, TENG can be designed to deal with various requirements [34–40]. In order to find a suitable TENG model, various structures have been studied, and three typical models that have been summarized are shown in Figure S4. Figure S4(i) shows a TENG supported by springs, which has poor controllability. In the process of controlled contact separation, the amplitude and the angle of shaking can significantly affect the output performance of TENG, which may hinder the combination of TENG and gas sensors. In Figure S4(ii), a disk-type TENG has been shown, which can produce a high output current. However, its matching resistance is out of the variation area of WO_3_. Figure S4(iii) takes the partial feather of an owl (see Figure S4(iv)) as the rotor to reduce the friction. However, the processing and assembling process of feathers is complicated, which makes the preparation more difficult and cost more. On the basis of these investigations, Figure 3(a) schematically illustrates the structure of the brush-shaped triboelectric nanogenerator (BS-TENG) consisting of the stator and the rotor. For the stator, a pair of Cu electrodes with complementary sectors is coated on a base plate through the printed circuit board (PCB) techniques (the angle of the Cu electrodes of 16°). For the rotor, the material of the friction layer is further investigated, as shown in Figure S5. After comparing the output voltage of fluorinated ethylene propylene (FEP) (0.05 mm), Polytetrafluoroethylene (PTFE) (0.05 mm), Kapton (0.65 mm), and Polyvinyl chloride (PVC) (0.1 mm) on the same stator, the FEP film with the highest output voltage has been selected as the dielectric material. In a real operation, FEP films have been negatively precharged by corona polarization, and the working principle of the BS-TENG mainly depends on electrostatic induction, as shown in Figure 3(a), (i)–(viii). To explain the detailed mechanism, only part of the BS-TENG has been exhibited in the diagrams (the state (i) is the initial state). When the precharged FEP film slides to the left (states (i)–(v)), electrons are driven from the left electrode to the right electrode, generating a current until the FEP film reaches the overlapping area of the next electrode (state (v)). After the FEP film passes another Cu electrode (states (v)–(viii)), the balance of the induced charge is disturbed again, causing electrons to flow in the opposite direction, leading to an inverted current. Then, for the consideration of wear resistance, the FEP film with a thickness of 0.1 mm has been used. The output voltage value is significantly increased by cutting the FEP film into 1 mm strip slices. Meanwhile, the number of stripes of the FEP films and the angle of electrodes can also affect the electrical performance of TENG. Figure S5b shows the output voltage of flat (FEP-0), single layer brush-shaped (FEP-1), double layer brush-shaped (FEP-2), 4-layer brush-shaped (FEP-4), and 6-layer brush-shaped (FEP-6) FEP films. It can be seen that the performance of the brush-like FEP is better than that of flat FEP. Meanwhile, the brush-shaped FEP film (FEP-2) with double layers has the best output effect in comparison with the others. The output voltage and current with load resistances of 5-5 (TENG's rotors have 5 double-layered brush-shaped FEP, and the electrode angle on the stator is 34°) and 10-10 (TENG's rotors have 10 double-layered brush-shaped FEP, and the electrode angle on the stator is 16°) under the rotating speed of 300 rpm is shown in Figure S5c. Figure S5d visually shows the voltage output parameters of flat FEP films and brush-like FEP films moving at the same speed under the same TENG structure. Brush-like FEP films are more beneficial to improve the output performance of TENG. Moreover, the FEP film surface before and after repeated friction shows little trace of wear (Figure S6). For the final device, ten FEP stripes (30 mm∗25 mm∗0.1 mm) are placed concentrically and uniformly on a round acrylic substrate.

Driving by a motor with a controller, the electric output of the BS-TENG has been characterized under different rotation speeds (see Figure 3(b)). In general, the voltage output of TENG is not related to the rotation rate but is decided by the maximum overlapped area of the two mediums. With the increase of the rotating speed, part of the FEP film leaves the stator substrate due to the centrifugal force, leading to the decrease of the contact area. Therefore, the slight decrease in voltage can be explained by the change of the contact area. Meanwhile, the output current is proportional to the rotating speeds as shown in Figure 3(c). The separation of the FEP film from the electrode at high rotating speed may also suppress the further increase of the current. Figure 3(d) exhibits the resistance dependence of the electric output with an external load from 0.1 M*Ω* to 40 M*Ω*, accompanied by the increase of load resistance, output voltage, and current change in opposite direction. Figure 3(e) shows the charging curves of BS-TENG as a power source; two capacitors are charged by the device with rectification. The large capacitor of 47 *μ*F can be charged to 25 V in 15 minutes from the initial state.

**Figure 4:**
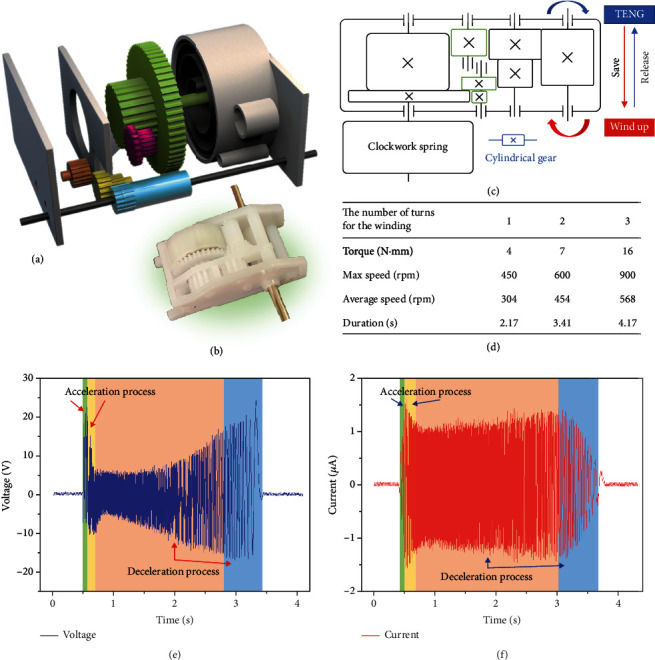
(a) With the increase of the number of turns of the winding, the related data is also gradually increased. Structural diagram of a pull back gearbox composed of 5 gears. (b) The photograph of the pull back gearbox. (c) The transmission diagram of the power gear set. (d) The torque of driven shafts, the TENG maximum, the average rpm, and the duration of each rotation. BS-TENG can reach a maximum speed of 900 rpm after being powered by a gearbox, with a continuous rotation rate of 4.17 s. The dynamic voltage (e) and current (f) curves of BS-TENG as it passes through the gear train.

In order to fabricate a fully portable and wireless gas sensor, the rotating motor for driving TENG needs to be replaced by some manual accelerator. Here, a pull back gearbox for providing high speed rotation is employed to drive TENG, as shown in Figure 4(a). The gearbox is mainly composed of 3 stationary gears, 2 movable gears, and 1 clockwork spring. The photographic diagram of the gearbox is shown in Figure 4(b), in which the shell size is approximately 30 mm∗13 mm∗18 mm. The shaft of TENG is fixed to the final gear in the gear system. Firstly, a human hand winds up the shaft to counterclockwise direction and the kinetic energy is stored in the clockwork spring. Then, when the hand moves away, the clockwork spring drives the shaft to rotate backward and the kinetic energy is released. The gears are designed to amplify the rotation speed, while the TENG connected with the final shaft can have a rather high rotating speed. Figure 4(c) is the transmission diagram of the power gear set. In this gear train, the gear ratio can be calculated as 0.286 based on the number of teeth of the first driving gear and the last driven gear (35 and 10, respectively). Green gears can be moved slightly in the process of energy storage and energy release in the gearbox, acting as the storage gear and transmission gear, respectively. During these two processes, different gears act as per their respective roles, as shown in Figure S7. In the process of energy saving, the human hand winds up through a red gear chain, which stores energy in the clockwork spring. When released, the clockwork spring drives the shaft through the blue gear chain. The process of the clockwork spring returning to its predeformed state is a process of nonuniform release of force, and thus, the gear driven by this force also presents a nonuniform state of force. The center of the rotor is fixed to the shaft on which the slave wheel is located, and the BS-TENG is indirectly driven by the gearbox, but the rotation trend of the BS-TENG disk is similar to the movement of gears. Figure S8 shows the photographic diagram and the outermost acrylic panel of the gas detector. With the increase of the number of turns of the winding, the torque of driven shafts, TENG's maximum average rotation speed (revolutions per minute, rpm), and duration of each rotation have gradually increased, as shown in Figure 4(d). BS-TENG can reach a maximum speed of 900 rpm after being driven by a gearbox, with a continuous rotating of 4.17 seconds, which provides sufficient time for subsequent signal conversion and output of BS-TENG from the gas sensor. The dynamic current and voltage curves of BS-TENG, driven by this gear accelerator without connecting the rectifier bridge, are shown in in Figure 4(e) and Figure 4(f). In the green and yellow areas of the figure, the current and voltage output of BS-TENG shows a trend of first increasing and then decreasing. It is because the clockwork spring deformation remains unchanged at the moment of release and the spring needs a certain time to gradually release the stored mechanical energy. Therefore, the motion state accelerates first, and then the acceleration decreases. During this green zone, the influence of the contact area of the FEP film on the voltage and current gradually increases until it is equal to the influence of the velocity on the voltage and current. In the yellow area, the contact area between the FEP film and the copper electrode decreases, making the electrical performance output smaller. Conversely, in the orange region of the deceleration process, the effect caused by the distance between the FEP film and the copper electrode prevailed. As the velocity slows down, the FEP film in the blue region recontacts with the copper electrode, the frictional resistance increases, and the rotation speed decreases rapidly. Therefore, the voltage and the speed increase slowly, and the current decreases rapidly.

**Figure 5:**
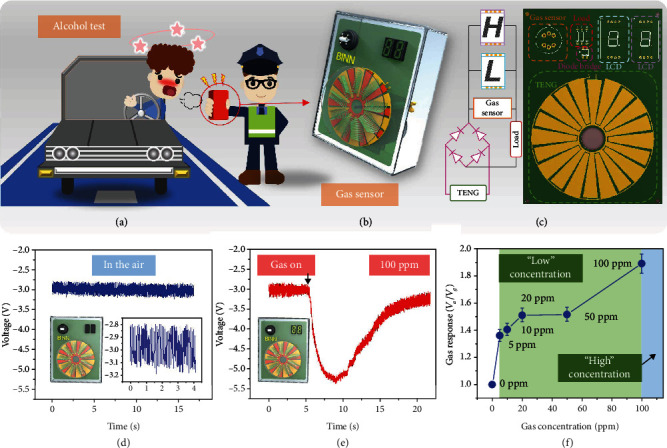
(a) The schematic diagram of the ethanol gas sensor used in the scene of traffic driving for alcohol detection. (b) The schematic diagram of an ethanol gas sensor. (c) The schematic diagram of the signal processing circuit and the location distribution diagram of the different components of the gas sensor on the PCB board. (d) At room temperature air conditions, the voltage drop at both ends of the WO_3_ sensor and the result of LCD. (e) The voltage change at both ends of the gas sensor when it comes into contact with 100 ppm of ethanol gas and the result of LCD. (f) The gas response curve of the ethanol detector at different gas concentrations, and the gas response is defined as the ratio *V*_*a*_/*V*_*g*_.

Unlike the alcohol sensors on the market that require supply power for a long time, a completely self-powered ethanol detector in the case of alcohol detection has been envisaged, as illustrated in Figure 5(a). To demonstrate that the matching performance of TENG and WO_3_ gas sensors, Figure 5(b) shows a diagram of an ethanol detector we designed. The BS-TENG is integrated with two signal-processing LCDs to display the real-time test results of the instrument. The circuit schematic diagram of the customized signal processing circuit and the wiring diagram of the PCB board are shown in Figure 5(c). Meanwhile, the distribution diagram of the actual PCB board is also shown in Figure 5(c), showing the distribution position of each component of the gas sensor on the PCB board. TENG has the characteristic of increasing the output voltage as the load resistance increases. Therefore, in the design of the circuit, a 250 M*Ω* fixed resistor is connected in series with the gas sensor. Then, two LCDs with customized operating voltages of 3 V and 5 V are connected in parallel to the gas sensors. At room temperature air conditions, the voltage drop at both ends of the WO_3_ sensor is almost zero during the rotation of BS-TENG, and the LCDs do not show any number. As the BS-TENG rotates, the 30 mL syringe filled with a certain concentration of ethanol gas is slowly released to the WO_3_ gas sensor. The ethanol gas significantly increases the resistance of the gas sensor. At the same time, TENG has the characteristic that the voltage at both ends increases with the increase of the resistance value of the external load resistance. Therefore, with the increase of the target gas concentration, the resistance at both ends of the gas sensor increases, and the voltage at both ends of the gas sensor increases. The first LCD can reach the working voltage when the concentration is low, and the second LCD reaches the working voltage when the concentration exceeds 100 ppm, as shown in Figure 5(e). In order to further test the reaction of the ethanol detector to gas detection, the circuit shown in Figure 5(c) has been used to test the detection of ethanol at different concentrations. The test results of real-time detection of voltage change at both ends of the sensor under 5-100 ppm atmosphere are shown in Figure S9. At concentrations of 5-50 ppm, the voltage drop on the gas sensor is larger than 3 V and the LCD showing “L” for “low concentration” is activated. Above 100 ppm, the voltage drop on the gas sensor is larger than 5 V and the LCD showing “H” for “high concentration” is turned on. At 50-100 ppm, the numbers on the two LCDs are unstable and flickering. Figure 5(f) shows the response curve of the ethanol detector at different gas concentrations, and the gas response is defined as the *V*_*a*_/*V*_*g*_, where *V*_*g*_ is the voltage in the atmosphere of ethanol while *V*_*a*_ is in an ethanol-free atmosphere. The video demonstration of this self-powered gas senor can be seen in the supporting information (see Movie 1).

## 3. Conclusion

In this work, a fully self-powered ethanol detector operating at room temperature has been developed and investigated, which mainly consisted of a BS-TENG, WO_3_ gas sensor, and two LCDs. WO_3_ nanorods for gas sensing exhibit good selectivity/anti-interference and durability after two weeks of storage in an air environment. BS-TENG matches the characteristic curve of voltage varying with external resistance to the effective detection range of the gas sensor by adjusting various structural parameters. The voltage drop of BS-TENG at both ends of the WO_3_ gas sensor is positively correlated with gas concentration, while the concentration change of ethanol in a range of 5-100 ppm can be distinguished by the output signal. Meanwhile, the gearbox with a clockwork spring has been added to support the continuous rotation of TENG for around 4 seconds. During this time, BS-TENG can power LCDs to visualize the results of ethanol concentration instantly. When ethanol gas is blown to the sensor, the ethanol detector can identify different concentrations and displays the result as “L” or “H” on the LCD, indicating the concentrations of 5-50 ppm (L) and the concentrations above 100 ppm (H). The gas detector based on BS-TENG and the concept of self-powered visualization present in this research can inspire a simple and widely applicable approach in the field of gas sensing.

## 4. Experimental Section

### 4.1. Fabrication of the BS-TENG

The BS-TENG consists of 3 parts: the stator, the rotator, and the gearbox. The stator of the TENG is a custom PCB board with a pair of Cu electrodes with complementary sectors which are coated on a base plate. The thickness is 1 mm. In the center of the PCB, there is a circle with a diameter of 16 mm, which serves as the fixed position of the drive shaft. The angle of the Cu electrodes is 16°. A round acrylic plate with a diameter of 80 mm and a thickness of 3 mm acts as the substrate for the rotor. A 2 mm round hole in the middle is used for fixing with the shaft. Ten FEP films (30 mm∗25 mm∗0.1 mm) are cut into 1 mm strips. Then, double-layer FEP strip films are placed concentrically and uniformly on the substrate. The polymer film of FEP is available in the market without further modification. The pull back gearbox comes from Vigor Toys Ltd. (VP131B).

### 4.2. Measurements of the Basic Performance of the BS-TENG

The voltage and current data are measured by a Keithley 6514 System Electrometer (LabVIEW program). The rotator of the BS-TENG is driven by a motor (SKISIA), and the rotation speed is measured by the laser contactless tachometer (UNI-T UT372). The torque measurement instrument is measured by DYN-200 dynamic torque sensor (Bengbu Dayang Sensing System Engineering Co., Ltd.).

### 4.3. WO_3_ Nanorod Synthesis and Characterization

The WO_3_ nanorods are prepared by hydrothermal process. Chemicals and materials can be used directly without further refinement. DI water is produced by the Millipore Direct-Q System. In a typical procedure, 3.30 g Na_2_WO_4_·2H_2_O and 1.17 g NaCl are dissolved in 75 mL of DI water. The pH of mixture is adjusted to 2.5 by HCl. And then, the mixture is transferred to the Teflon-lined stainless steel autoclave and kept at 180°C for 24 h. The mixture is then cooled naturally and washed alternately with DI water and ethanol. The filtered solid powder of WO_3_ is then dried at 60°C for 2 h. The microscopic morphology of the WO_3_ is given by a scanning SU8020 cold-field SEM. The elemental analysis is given by EDS of the SU8020 microscope. The typical WO_3_ nanorods are characterized by XRD (Bruker D8 Advance).

### 4.4. Assembly of Gas Sensors and Gas-Sensing Measurement

In the process of making the gas sensor, an appropriate amount of WO_3_ nanorods is first added to the agate pot and dropped into a little DI water to be uniformly ground and form a slurry. Then, the slurry is painted on a 1 mm diameter 4 mm earthenware tube. Finally, the earthenware tube is soldered to the base. The basic consumables for gas sensors come from Winsen Technology.

The gas-sensitive measurement is carried out in a 2.5 L enclosed acrylic container. Liquid ethanol is injected into a PTC thermostatic heater at 75°C. The gas ethanol is filled dynamically in the container. The resistance change is measured by the Keithley 6517 digital multimeter (Victor 990C+). When the output value is stable, the test box is opened and the sensor returns to normal in the air.

## Data Availability

The electrical data, torque data, and resistance data used to support the findings of this study are included within the article and the supplementary information file. The X-ray diffraction data, EDS, and customized design drawing of LCD, used to support the findings of this study, are available from the corresponding author upon request.

## Supplementary Materials

Supplementary Materials Figure S1: SEM images of the WO3 obtained at pH = 1.7, 1.9, 2.1, 2.3, and 2.5. Under different pH values, the hydrothermal reaction can obtain WO_3_ is obtained by with diverse morphologies. Figure S2: the EDS analysis of WO_3_. Figure S3: after two weeks of natural storage at room temperature, the response resistance of the WO_3_ gas sensor from 35 to 20 M*Ω*. Figure S4:three TENG models have been tried, vertical contact-separation mode (i) and rotating disk mode (ii); the owl's feathers act as a friction material. Figure S5: for the rotor, the material of the friction layer is investigated. Figure S6: the FEP film surface before and after repeated friction shows little trace of wear. Figure S7: the transmission diagram of the gearbox. The red gears on the left are involved in storing energy. The blue gear on the right is involved in the energy release process. Figure S8: the photographic diagram, the outermost acrylic panel of the gas detector, and the rotor of BS-TENG. Figure S9: the schematic diagram of the device for detecting 5 ppm ethanol gas and the voltage reaction of the alcohol sensor to different concentrations from 5 to 100 ppm. Movie 1: the video demonstration of a self-powered gas senor.
