# Supplementary material for: Self-Powered Room-Temperature Ethanol Sensor Based on Brush-Shaped Triboelectric Nanogenerator
Source: Research (Wash D C). 2021 Mar 1;2021:8564780. doi: 10.34133/2021/8564780 (PMC7945684; doi:10.34133/2021/8564780)
Supplement: Supplementary Materials — Figure S1: SEM images of the WO3 obtained at pH = 1.7, 1.9, 2.1, 2.3, and 2.5. Under different pH values, the hydrothermal reaction can obtain WO3 is obtained by with diverse morphologies. Figure S2: the EDS analysis of WO3. Figure S3: after two weeks of natural storage at room temperature, the response resistance of the WO3 gas sensor from 35 to 20 MΩ. Figure S4:three TENG models have been tried, vertical contact-separation mode (i) and rotating disk mode (ii); the owl's feathers act as a friction material. Figure S5: for the rotor, the material of the friction layer is investigated. Figure S6: the FEP film surface before and after repeated friction shows little trace of wear. Figure S7: the transmission diagram of the gearbox. The red gears on the left are involved in storing energy. The blue gear on the right is involved in the energy release process. Figure S8: the photographic diagram, the outermost acrylic panel of the gas detector, and the rotor of BS-TENG. Figure S9: the schematic diagram of the device for detecting 5 ppm ethanol gas and the voltage reaction of the alcohol sensor to different concentrations from 5 to 100 ppm. Movie 1: the video demonstration of a self-powered gas senor. [file 8564780.f1.zip › Supporting Information.pdf]

Supporting Information

Self-powered room-temperature ethanol sensor based on brush-shape triboelectric nanogenerator

Jingwen Tian [ab], *Fan Wang* [ab], *Yafei Ding* [ab], *Rui Lei* [ab], *Yuxiang Shi* [ab], *Xinglin Tao* [ab], *Shuyao Li* [ab], *Ya Yang** [ab], *Xiangyu Chen** [ab]

[a] CAS Center for Excellence in Nanoscience, Beijing Key Laboratory of Micro-nano Energy and Sensor, Beijing Institute of Nanoenergy and Nanosystems, Chinese Academy of Sciences, Beijing 100083, China

[b] School of Nanoscience and Technology, University of Chinese Academy of Sciences, Beijing 100049, China

E-mail: chenxiangyu@binn.cas.cn, yayang@binn.cas.cn

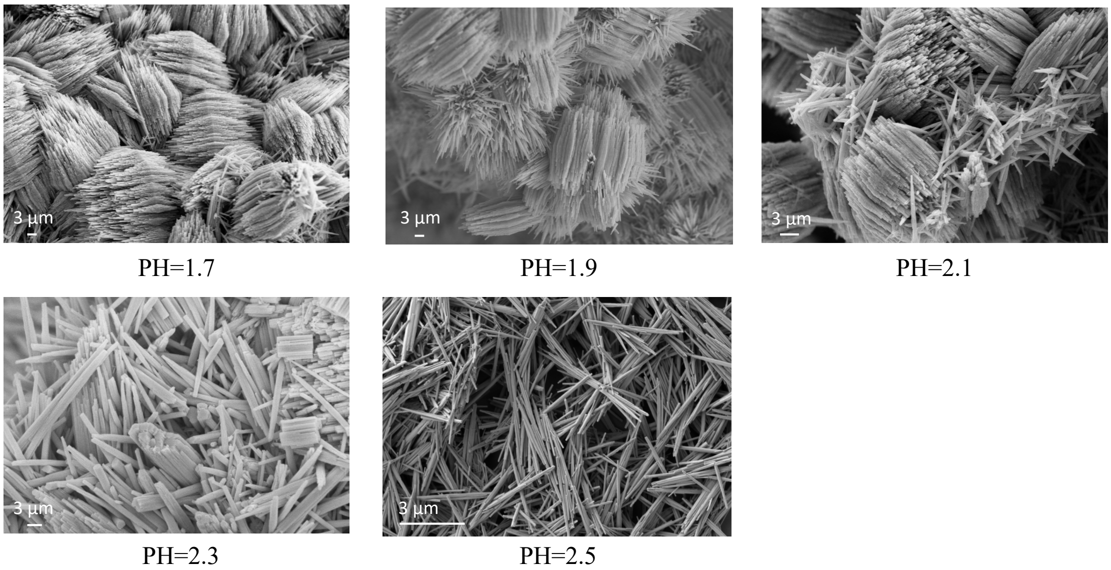

Figure S1. SEM images of the WO_3 obtained at pH = 1.7, 1.9, 2.1, 2.3, and 2.5. Under different PH values, the hydrothermal reaction can obtain WO_3 is obtained by with diverse morphologies.

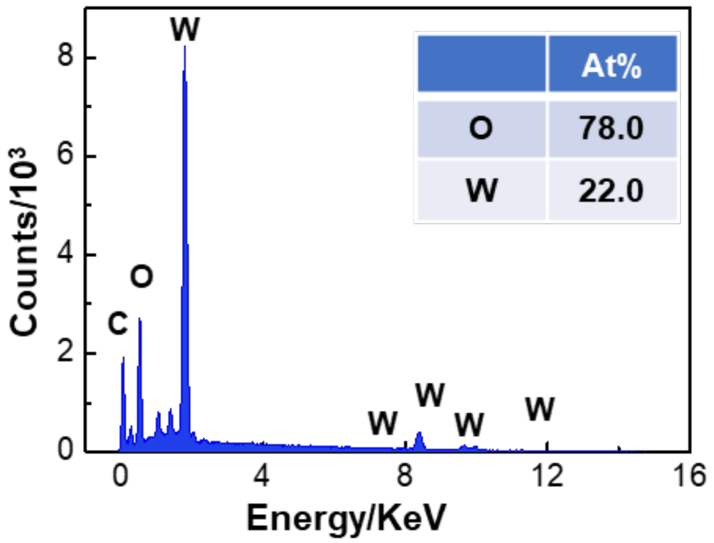

Figure S2. The EDS analysis of WO_3.

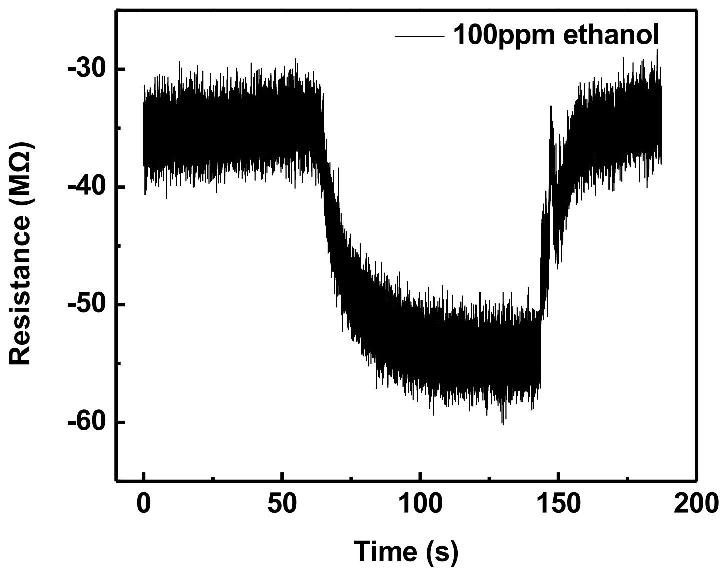

Figure S3. After two weeks of natural storage at room temperature, the respond resistance of WO_3 gas sensor from 35 to 20 MΩ.

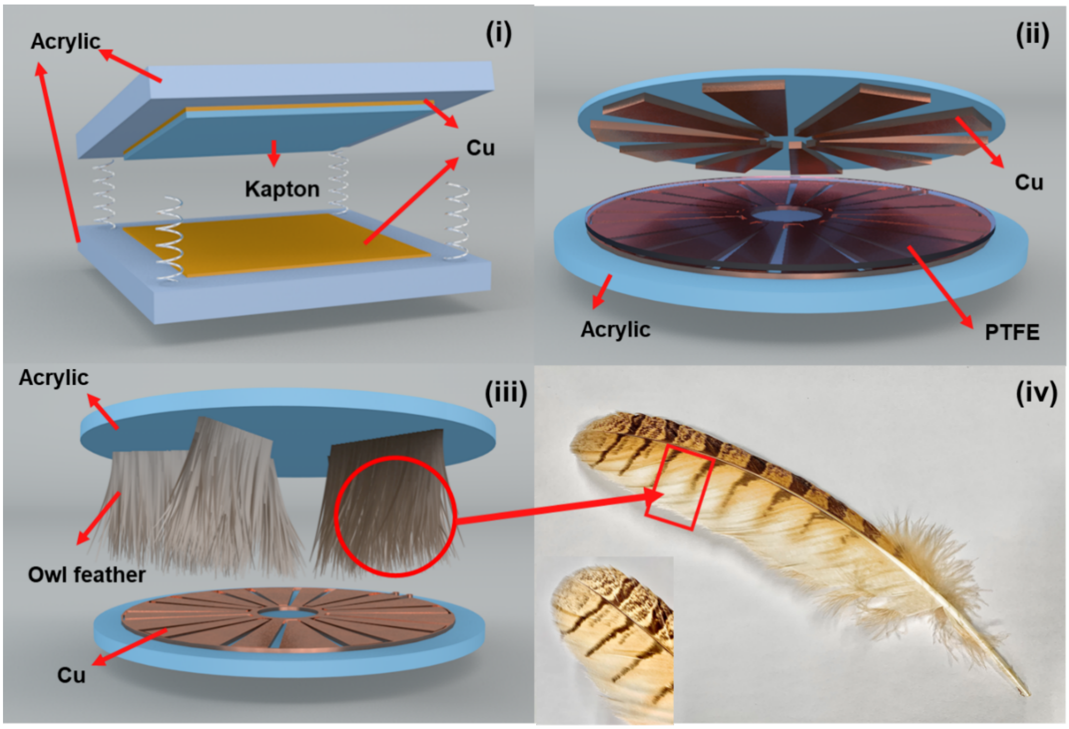

Figure S4. Three TENG models has been tried, vertical contact-separation mode(i), rotating disk mode (ii), the owl's feathers act as friction material.

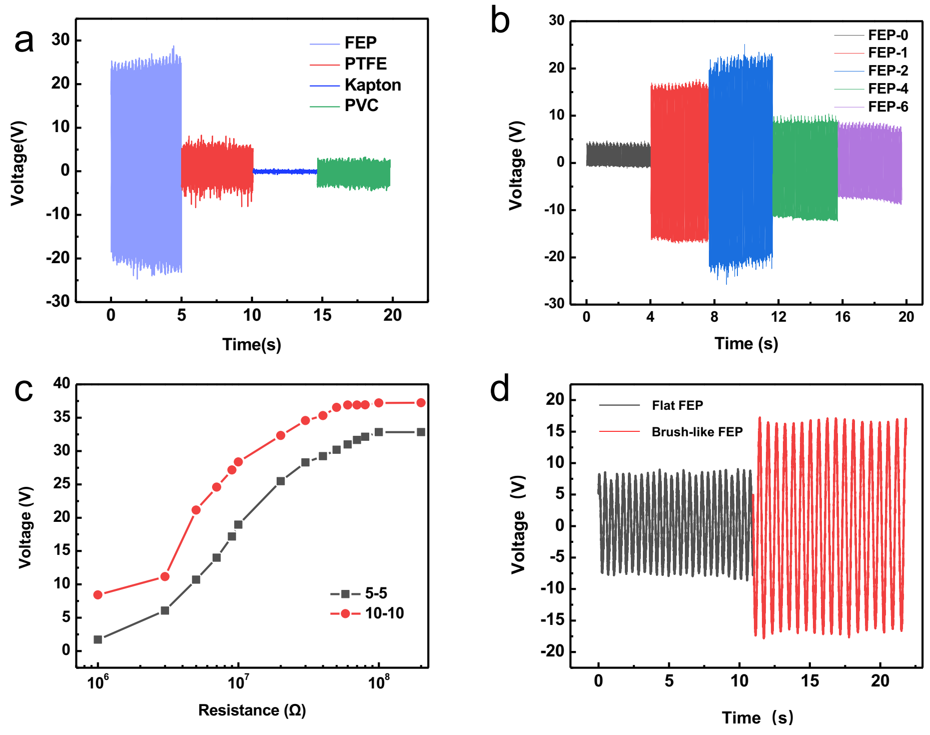

Figure S5. For the rotor, the material of the friction layer is investigated. (a) The output voltage of fluorinated ethylene propylene (FEP) (0.05mm), PTFE (0.05mm), Kapton (0.65mm), and PVC (0.1mm) on the same stator. (b) The output voltage of sheet FEP film (FEP-0), single layer brush-shape (FEP-1), double layer brush-shape (FEP-2), 4-layer brush-shape (FEP-4) and 6-layer brush-shape (FEP-6) FEP film. (c) The output voltage and current with external load resistances of 5-5 (TENG's rotors have 5 double-layered brush-shape FEP, and the electrode angle on the stator is 34˚). and 10-10 (TENG's rotors have 10 double-layered brush-shape FEP, and the electrode angle on the stator is 16˚) under the rotating speed of 300 rpm. (d) At a speed of 16 rpm, the respective voltage outputs of plat FEP and brush-like FEP on the same TENG structure.

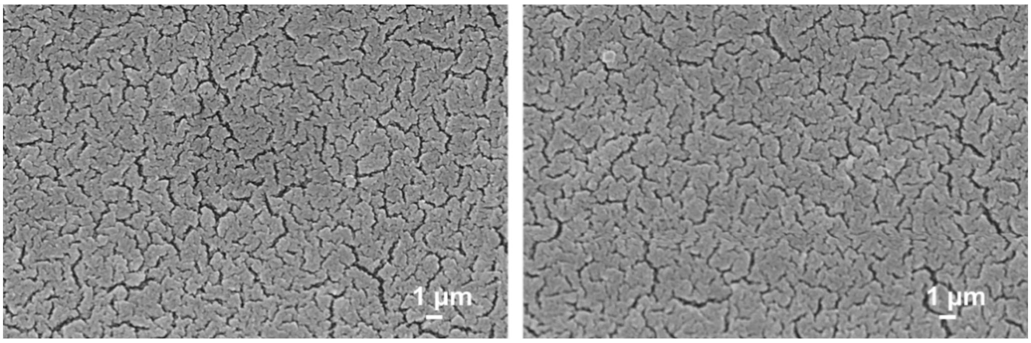

Figure S6. The FEP film surface before and after repeated friction shows little trace of wear.

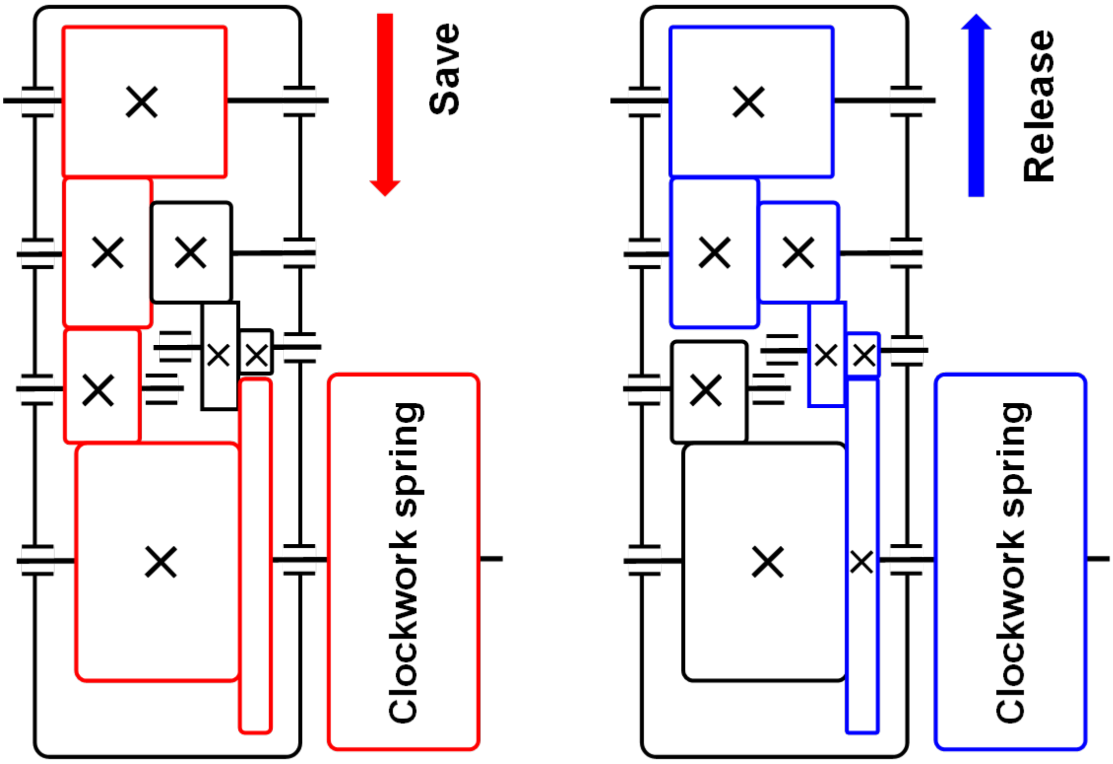

Figure S7. The transmission diagram of gearbox. The red gears on the left are involved in storing energy. The blue gear on the right is involved in the energy release process.

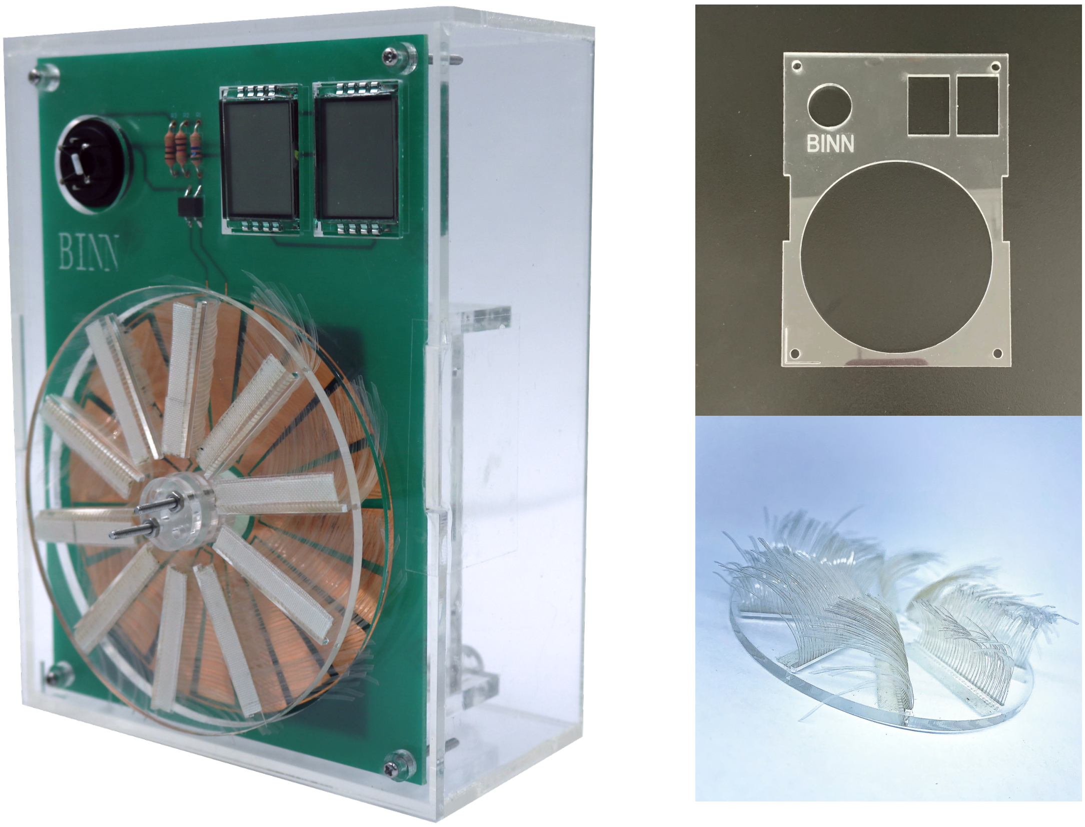

Figure S8. The photographic diagram, the outermost acrylic panel of the gas detector and the rotor of BS-TENG.

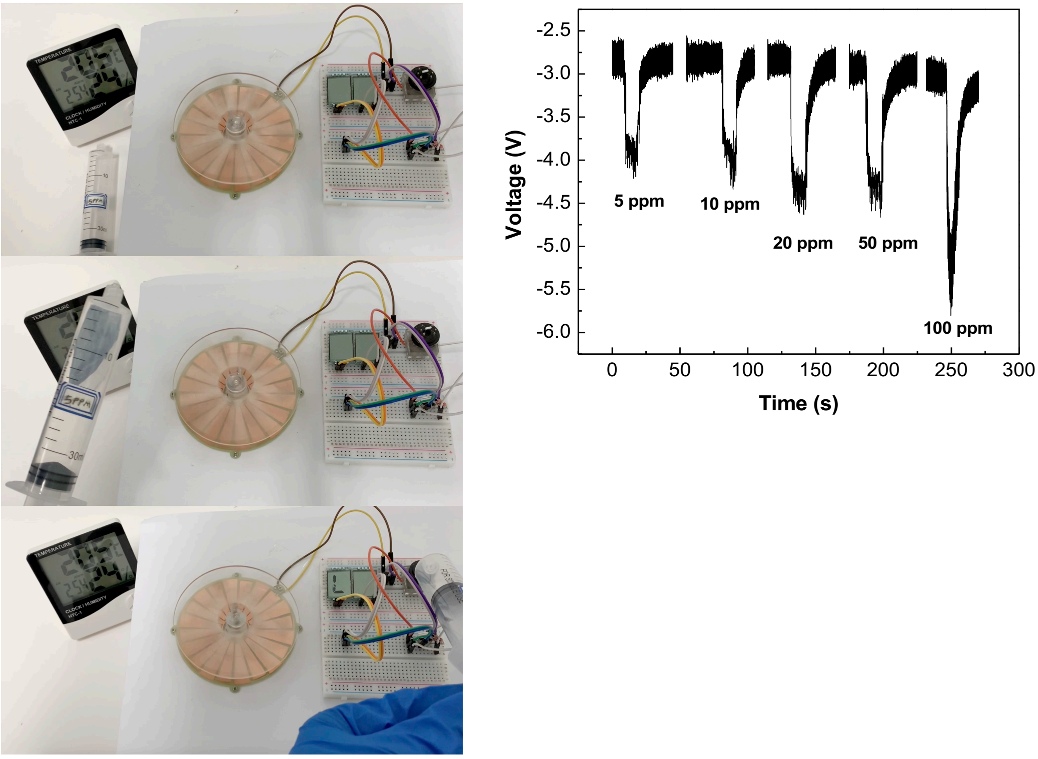

Figure S9. The schematic diagram of device for detecting 5ppm ethanol gas and the voltage reaction of the alcohol sensor to different concentration from 5 to 100ppm.
