# Supplementary material for: Self-Powered Room-Temperature Ethanol Sensor Based on Brush-Shaped Triboelectric Nanogenerator
Source: Research (Wash D C). 2021 Mar 1;2021:8564780. doi: 10.34133/2021/8564780 (PMC7945684; doi:10.34133/2021/8564780)
Supplement: Supplementary Materials — Figure S1: SEM images of the WO3 obtained at pH = 1.7, 1.9, 2.1, 2.3, and 2.5. Under different pH values, the hydrothermal reaction can obtain WO3 is obtained by with diverse morphologies. Figure S2: the EDS analysis of WO3. Figure S3: after two weeks of natural storage at room temperature, the response resistance of the WO3 gas sensor from 35 to 20 MΩ. Figure S4:three TENG models have been tried, vertical contact-separation mode (i) and rotating disk mode (ii); the owl's feathers act as a friction material. Figure S5: for the rotor, the material of the friction layer is investigated. Figure S6: the FEP film surface before and after repeated friction shows little trace of wear. Figure S7: the transmission diagram of the gearbox. The red gears on the left are involved in storing energy. The blue gear on the right is involved in the energy release process. Figure S8: the photographic diagram, the outermost acrylic panel of the gas detector, and the rotor of BS-TENG. Figure S9: the schematic diagram of the device for detecting 5 ppm ethanol gas and the voltage reaction of the alcohol sensor to different concentrations from 5 to 100 ppm. Movie 1: the video demonstration of a self-powered gas senor. [file 8564780.f1.zip › toc.pdf]

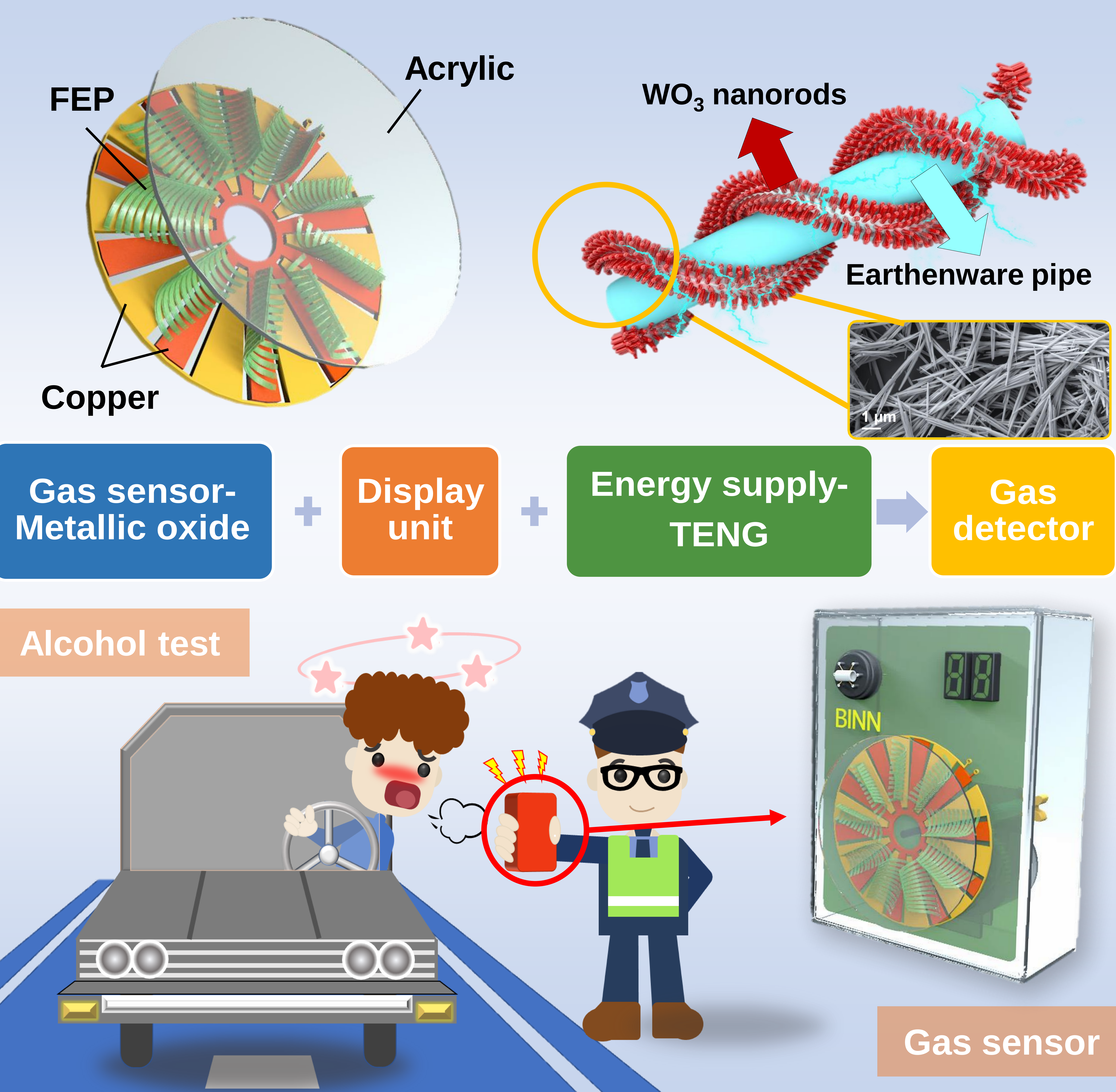

FEP
Acrylic
Copper
WO_3 nanorods
Earthenware pipe
1 μm
Gas sensor-
Metallic oxide
+
Display
unit
+
Energy supply-
TENG
Gas
detector
Alcohol test
BINN
Gas sensor
